# A time-adjusted control chart for monitoring surgical outcome variations

**DOI:** 10.1371/journal.pone.0303543

**Published:** 2024-05-15

**Authors:** Quentin Cordier, My-Anh Le Thien, Stéphanie Polazzi, François Chollet, Matthew J. Carty, Jean-Christophe Lifante, Antoine Duclos

**Affiliations:** 1 Research on Healthcare Performance RESHAPE, INSERM U1290, Université Claude Bernard Lyon 1, Lyon, France; 2 Health Data Department, Hospices Civils de Lyon, Lyon, France; 3 Center for Surgery and Public Health, Brigham and Women’s Hospital, Harvard Medical School, Boston, Massachusetts, United States of America; 4 Hospices Civils de Lyon, Centre Hospitalier Lyon Sud, Service de Chirurgie Générale et Endocrinienne, Pierre Bénite, France; E-Da Cancer Hospital, TAIWAN

## Abstract

**Background:**

Statistical Process Control (SPC) tools providing feedback to surgical teams can improve patient outcomes over time. However, the quality of routinely available hospital data used to build these tools does not permit full capture of the influence of patient case-mix. We aimed to demonstrate the value of considering time-related variables in addition to patient case-mix for detection of special cause variations when monitoring surgical outcomes with control charts.

**Methods:**

A retrospective analysis from the French nationwide hospital database of 151,588 patients aged 18 and older admitted for colorectal surgery between January 1^st^, 2014, and December 31^st^, 2018. GEE multilevel logistic regression models were fitted from the training dataset to predict surgical outcomes (in-patient mortality, intensive care stay and reoperation within 30-day of procedure) and applied on the testing dataset to build control charts. Surgical outcomes were adjusted on patient case-mix only for the classical chart, and additionally on secular (yearly) and seasonal (quarterly) trends for the enhanced control chart. The detection of special cause variations was compared between those charts using the Cohen’s Kappa agreement statistic, as well as sensitivity and positive predictive value with the enhanced chart as the reference.

**Results:**

Within the 5-years monitoring period, 18.9% (28/148) of hospitals detected at least one special cause variation using the classical chart and 19.6% (29/148) using the enhanced chart. 59 special cause variations were detected overall, among which 19 (32.2%) discordances were observed between classical and enhanced charts. The observed Kappa agreement between those charts was 0.89 (95% Confidence Interval [95% CI], 0.78 to 1.00) for detecting mortality variations, 0.83 (95% CI, 0.70 to 0.96) for intensive care stay and 0.67 (95% CI, 0.46 to 0.87) for reoperation. Depending on surgical outcomes, the sensitivity of classical versus enhanced charts in detecting special causes variations ranged from 0.75 to 0.89 and the positive predictive value from 0.60 to 0.89.

**Conclusion:**

Seasonal and secular trends can be controlled as potential confounders to improve signal detection in surgical outcomes monitoring over time.

## Background

Adverse events following surgery remain frequent, resulting in one out of ten patients being exposed to severe and preventable harm [1]. As a solution, surgical outcomes monitoring over time has proven impactful for reducing patient mortality and morbidity [2]. To avoid misinterpretation in assessing outcomes variation prospectively, indicators need to be adjusted for patient case-mix. However, data granularity extracted from available data sources does not fully capture every patient nuance or surgical procedure complexity. Hospital data warehouse accuracy is also uncertain because information is not primarily collected for quality improvement purposes [3]. Data validity can be heterogeneous across hospitals, resulting in flawed interpretation of their performance [4, 5].

To deal with these methodological challenges, considering time-related proxies such as secular and seasonal trends could be of particular interest when tracking surgical safety. In past control chart literature, these variables were not identified as critical for surgical outcomes adjustment [6]. Secular trends reflect enhancing or deteriorating outcomes over years nationwide under the influence of a myriad of drivers [7]. Over seasons, patient case-mix also fluctuates with a marked volume reduction in elective procedures and more complex cases performed during the summer holidays [8, 9]. Therefore, integrating annual secular trends and quarterly seasonal variations in the adjustment scheme may indirectly control variations related to both patient severity and data validity that may influence outcome irrespective of the delivered surgical quality [10].

The Shewhart control chart is increasingly used for healthcare improvement, providing a visual presentation of data easily interpretable by healthcare professionals. This decision support tool plots successive indicator measurements in chronological order, with control limits demarcating their expected variation [11]. Validated through a century of usage in industry, previous experience suggested its transferability to improve healthcare quality [12]. We assumed that adjusting control charts for time-related variables would change the interpretation of indicator variations and refine the detection of outliers in surgical outcomes. The present study aimed to compare the detection of special cause variations in colorectal surgery using an enhanced time-adjusted control chart adjusted for secular and seasonal trends in addition to the patient case-mix versus a classical control chart accounting for patient case-mix only.

## Methods

### Study population, outcomes and design

**Fig 1:**
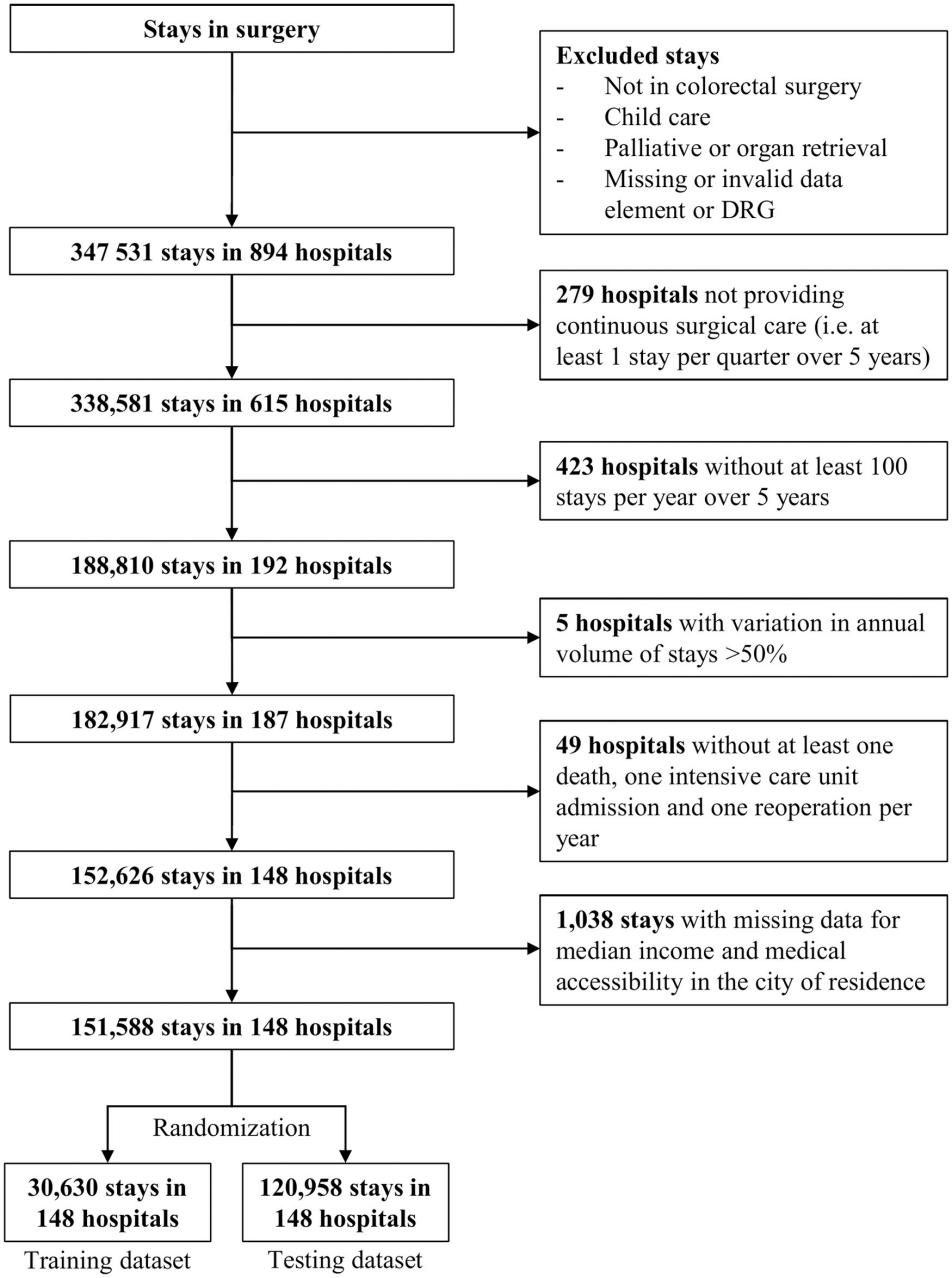
Study flowchart.

We included all stays for patients aged 18 and older admitted to French public or private hospitals for colorectal surgery between January 1^st^, 2014, and December 31^st^, 2018. Inpatient stays for palliative care or organ retrieval were excluded, as well as hospitals providing colorectal surgery discontinuously (less than 1 stay per quarter over 5 years), with low volume of stays for colorectal surgery (less than 100 stays per year over 5 years), with important variations in their annual volume of stays (variation greater than 50%), or without at least one occurrence of each monitored outcome per year, allowing us to build charts with sufficient volume of procedures per quarter in each hospitals along the 5 years study. Considering their low frequency, patients with missing data were also excluded (Fig 1).

The three monitored outcomes on control charts were major adverse events occurring within 30 days from the colorectal surgical procedure, categorized according to the Dindo-Clavien classification, which ranks a surgical complication in an objective and reproducible manner [13]. Those outcomes included inpatient death, intensive care stay (at least two nights in intensive care unit or five nights in intermediate care unit), and reoperation (open or laparoscopic digestive tract procedure).

To fit models for outcome adjustment independently from the data to be monitored on control charts, we randomly divided our dataset into a training set and a testing set with stratification by hospital and the period for every quarter per year [14, 15]. The training set accounted for 20% of the data (30,630 stays) and the testing set for the 80% remaining (120,958 stays). The choice of 20/80 splitting implied that the study sample was large enough to build reliable models using the training set first, then allowing us to use a majority share of the data to build the charts from the testing set. Classical control charts were adjusted for patient case-mix only, while enhanced control charts were adjusted with the same set of variables and the additional input of secular (yearly) and seasonal (quarterly) trends. Finally, the detection of quarterly special cause variation signals between those charts was compared.

### Data source

Anonymised data were obtained from the French Medical Information System (Programme de Médicalisation des Systèmes d’Information (PMSI), source: ATIH [16]). This nationwide database is used routinely for care reimbursement and is updated weekly with data collected prospectively from all hospitals in France. Due to the accuracy and exhaustive data collection of the PMSI database, no patients were lost to follow-up during the study period. Inpatient stays were recorded as standard discharge abstracts containing compulsory information about patients and their primary or secondary diagnoses using ICD-10 (International Classification of Diseases, 10th revision) codes as well as detailed procedural codes associated with the care provided. Patients’ socioeconomic status (median household income in the city of residence) and medical accessibility (mean number of family medicine consultations per year and per inhabitant in the city of residence) were retrieved from their city of residence postcode. The study was conducted in accordance with the guidelines of the Declaration of Helsinki and recorded by the French National Data Protection Commission (CNIL, MR005 N° MR0612180919). It was strictly observational, relying on anonymous data available to investigators through the Secured Data Access Platform of the French Technical Agency of Hospital Information under formal authorization and a secure process. Consequently, according to French law, the need for written consent was waived by the CNIL, and no institutional review board approval was necessary.

### Statistics and charts

Multilevel logistic regression models were fitted from the training set using Generalized Estimating Equations (GEEs, see S1 Supporting information in S1 File) [17] for parameters associated with the three outcomes, with an exchangeable working correlation structure to account for clustering of patients within each hospital [18]. Classical control charts models were adjusted for case-mix variables only including: age, gender, patients’ socioeconomic status, medical accessibility, emergency admission, hospital status, surgical procedure complexity (segmental colectomy, total colectomy, coloproctectomy, rectopexy, rectal surgery, ostomy and Hartmann procedure reversal), primary diagnosis (cancer, diverticulosis, inflammatory bowel disease, bowel occlusion, ostomy surveillance, other surgical indication) and comorbidities (in dummy variables) as determined by the Elixhauser score [19]. Enhanced case-mix and time-adjusted control charts models were adjusted with the same set of variables, in addition to the year as a proxy for secular trends and the quarter as a proxy of seasonal variations. Model calibration was assessed by plotting the observed frequency of events per tenths of predicted risk [20], and model discrimination was evaluated using c-statistics (or area under the curve AUC).

We retained the regression estimates and the intraclass correlation coefficient (ICC) to determine the design effect of each hospital and reflect the inflation in variance due to the clustering of patients within the same hospital. Then, using regression estimates obtained from the training dataset, we computed for each operated patient of the testing dataset the expected probability of each outcome.

Shewhart p-control charts were designed to monitor outcomes within each hospital using the testing dataset. A control chart is a graphical tool used to monitor the evolution of a statistical process compared to a central line, upper and lower control limits. It enables its user to determine whether the observed value falls within the control limits, indicating that the process is under control. Conversely, if the observed value is beyond the control limits, the process is considered out of control, signaling the presence of a special cause variation [21]. As our outcomes were binary, we have opted for an adapted form of control charts specifically designed for binary indicators, the Shewhart p-control chart. Each data point on the chart represented the crude proportion of the observed outcome per quarter, accounting for a total of 20 points over the five years monitoring period. The central line value of the charts was not constant and represented the expected proportion of complications per quarter calculated from the models initially fitted on the training dataset and then applied to the testing dataset. Exact control and warning limits were set at 3 SD (99.73%) and 2 SD (95.45%) from the central line respectively using the exact binomial distribution [11].

We additionally corrected these limits through the intra-class correlation coefficient to account for the inflation of variance due to the pooling of patients within a same hospital [18]. Finally, in order to avoid inter-hospital hospital benchmarking and allow the accurate detection of special cause variations within the same hospital, we recalibrated the central line and limits depending on the overall observed rates per hospital. In cases where calculated values were beyond 1 or below 0 (values that are conceptually impossible), such limits were reset to 1 and 0, respectively. Formulas used to build the charts are available in S2 Supporting information in S1 File). The detection of special cause variation signals was defined as a single point outside the 3 SD control limits or two out of three successive points between the 2 SD warning limit and the 3 SD control limit on the same side of the central line. Since the risk of detecting false-positive special cause variation increases as the number of detection increases, we restricted our detection methodology to these two rules, corresponding to an overall 1/225 (0.444%) risk of false positive per plotted value [22], and an 1-(1–0.444)^20^ = 8.5% risk of detecting at least one false-positive signal in a 20-points control chart [23].

Following the chart construction, an agreement table for the detection of special cause variations was set up between the classical control charts and the enhanced control charts for each monitored outcome among all hospitals (148 hospitals x 20 quarters = 2,960 points in total). Agreement measure was computed using Cohen’s Kappa statistic [24] with corresponding 95% CI. Agreement in signals detection of special cause variations was considered acceptable above 0.60 and good above 0.80 [25].

Finally, considering the enhanced control chart as the reference, we computed sensitivity and positive predictive values of the classical control chart to detect valid special cause variation.

Data manipulation and analyses were performed using SAS software (version 9.4; SAS Institute Inc., Cary, NC).

## Results

A total of 151,588 inpatient stays for colorectal surgery performed in 148 hospitals were considered in our analyses. Inpatient stay characteristics per quarter are described in Table 1, revealing higher risk and poorer outcomes among patients who underwent surgery during the third quarter. Patients treated during this period were significantly older (mean age of 65.4 versus 64.9 for the whole year) with a greater share of males (50.3% versus 48.8%), higher Elixhauser comorbidity scores (mean score of 1.6 versus 1.5) than patients treated in other quarters. They were also more often admitted emergently (14.4% versus 13.6%) with cancer diagnoses (55.9% versus 52.3%). Among the monitored adverse events, inpatient mortality was significantly higher during the third quarter (4.2% versus 3.9% for the whole year), as were rates of intensive care stay (9.9% versus 9.2%) and reoperation (9.4% versus 8.9%).

**Table 1:** Inpatient stays characteristics per quarter.

Patient stays characteristics	Quarter				Total N = 151 588	P-value
	Q1 (N = 39 104)	Q2 (N = 38 661)	Q3 (N = 35 060)	Q4 (N = 38 763)		
Age, mean (std)	64.6 (15.0)	65.0 (15.1)	65.4 (15.2)	64.8 (15.2)	64.9 (15.1)	<0.001
Male, N (%)	18 845 (48.2%)	18 848 (48.8%)	17 630 (50.3%)	18 707 (48.3%)	74 030 (48.8%)	<0.001
Median household income in the city of residence in K€, mean (std)	21.1 (3.6)	21.1 (3.6)	21.1 (3.6)	21.1 (3.6)	21.1 (3.6)	0.386
Medical accessibility, mean (std)	4.2 (1.2)	4.2 (1.2)	4.2 (1.2)	4.2 (1.2)	4.2 (1.2)	0.001
Hospital status, N (%)						0.334
*University hospital (41 hospitals)*	14 027 (35.9%)	13 944 (36.1%)	12 738 (36.3%)	14 014 (36.2%)	54 723 (36.1%)	
*Private (56 hospitals)*	14 175 (36.2%)	13 863 (35.9%)	12 404 (35.4%)	13 741 (35.4%)	54 183 (35.7%)	
*Public (51 hospitals)*	10 902 (27.9%)	10 854 (28.1%)	9918 (28.3%)	11 008 (28.4%)	42 682 (28.2%)	
Emergency admission, N (%)	5171 (13.2%)	5090 (13.2%)	5039 (14.4%)	5274 (13.6%)	20 574 (13.6%)	<0.001
Main diagnosis, N (%)						<0.001
*Cancer*	19 469 (49.8%)	20 433 (52.9%)	19 594 (55.9%)	19 746 (50.9%)	79 242 (52.3%)	
*Other* *	8725 (22.3%)	8177 (21.2%)	6792 (19.4%)	8372 (21.6%)	32 066 (21.2%)	
*Diverticulosis*	5703 (14.6%)	5002 (12.9%)	4322 (12.3%)	5521 (14.2%)	20 548 (13.6%)	
*Ostomy surveillance*	2386 (6.1%)	2310 (6.0%)	1842 (5.3%)	2184 (5.6%)	8722 (5.8%)	
*Bowel occlusion*	1479 (3.8%)	1419 (3.7%)	1377 (3.9%)	1487 (3.8%)	5762 (3.8%)	
*Inflammatory bowel disease*	1342 (3.4%)	1320 (3.7%)	1133 (3.2%)	1453 (3.7%)	5248 (3.5%)	
Surgical procedure, N (%)						<0.001
*Segmental colectomy*	21 910 (56.0%)	21 767 (56.3%)	20 434 (58.3%)	21 771 (56.2%)	85 882 (56.7%)	
*Rectal surgery*	9233 (23.6%)	9523 (24.6%)	8705 (24.8%)	9540 (24.6%)	37 001 (24.4%)	
*Rectopexy*	3882 (9.9%)	3435 (8.9%)	2478 (7.1%)	3579 (9.2%)	13 374 (8.8%)	
*Ostomy and Hartmann procedure reversal*	2231 (5.7%)	2181 (5.6%)	1780 (5.1%)	2078 (5.4%)	8270 (5.5%)	
*Total colectomy or coloproctectomy*	1848 (4.7%)	1755 (4.5%)	1663 (4.7%)	1795 (4.6%)	7061 (4.7%)	
Elixhauser, mean (std)	1.5 (1.7)	1.5 (1.7)	1.6 (1.7)	1.5 (1.7)	1.5 (1.7)	<0.001
AIDS/HIV	57 (0.1%)	63 (0.2%)	60 (0.2%)	63 (0.2%)	243 (0.2%)	0.8317
Alcohol abuse	865 (2.2%)	812 (2.1%)	861 (2.5%)	831 (2.1%)	3369 (2.2%)	0.0073
Anemia (blood loss)	695 (1.8%)	707 (1.8%)	595 (1.7%)	653 (1.7%)	2650 (1.7%)	0.4065
Anemia (deficiency)	1333 (3.4%)	1364 (3.5%)	1265 (3.6%)	1378 (3.6%)	5340 (3.5%)	0.5732
Cardiac arrhythmias	4558 (11.7%)	4452 (11.5%)	4266 (12.2%)	4504 (11.6%)	17780 (11.7%)	0.0371
Chronic pulmonary disease	2234 (5.7%)	2111 (5.5%)	1968 (5.6%)	2137 (5.5%)	8450 (5.6%)	0.4467
Coagulopathy	763 (2.0%)	750 (1.9%)	667 (1.9%)	741 (1.9%)	2921 (1.9%)	0.9590
Congestive heart failure	1828 (4.7%)	1821 (4.7%)	1759 (5.0%)	1881 (4.9%)	7289 (4.8%)	0.1618
Depression	1133 (2.9%)	1185 (3.1%)	1062 (3.0%)	1146 (3.0%)	4526 (3.0%)	0.5285
Diabete with chronic complication	737 (1.9%)	686 (1.8%)	719 (2.1%)	712 (1.8%)	2854 (1.9%)	0.0283
Diabete without chronic complication	3632 (9.3%)	3756 (9.7%)	3445 (9.8%)	3590 (9.3%)	14423 (9.5%)	0.0158
Drug abuse	79 (0.2%)	88 (0.2%)	80 (0.2%)	71 (0.2%)	318 (0.2%)	0.4993
Fluid and electrolyte disorders	4761 (12.2%)	4578 (11.8%)	4487 (12.8%)	4773 (12.3%)	18599 (12.3%)	0.0049
Hemiplegia or paraplegia	413 (1.1%)	426 (1.1%)	374 (1.1%)	415 (1.1%)	1628 (1.1%)	0.9493
Hypertension, complicated	351 (0.9%)	319 (0.8%)	303 (0.9%)	297 (0.8%)	1270 (0.8%)	0.1977
Hypertension, uncomplicated	10355 (26.5%)	10345 (26.8%)	9672 (27.6%)	10104 (26.1%)	40476 (26.7%)	0.0004
Hypothyroidism	1384 (3.5%)	1331 (3.4%)	1196 (3.4%)	1390 (3.6%)	5301 (3.5%)	0.5381
Liver disease	987 (2.5%)	1006 (2.6%)	950 (2.7%)	1046 (2.7%)	3989 (2.6%)	0.3754
Lymphoma	195 (0.5%)	173 (0.4%)	178 (0.5%)	213 (0.5%)	759 (0.5%)	0.2075
Metastatic solid tumor	4560 (11.7%)	4699 (12.2%)	4565 (13.0%)	4489 (11.6%)	18313 (12.1%)	<0.001
Obesity	3316 (8.5%)	3226 (8.3%)	2974 (8.5%)	3243 (8.4%)	12759 (8.4%)	0.8746
Other neurological disorders	849 (2.2%)	800 (2.1%)	704 (2.0%)	807 (2.1%)	3160 (2.1%)	0.4892
Peptic ulcer disease	67 (0.2%)	58 (0.2%)	59 (0.2%)	65 (0.2%)	249 (0.2%)	0.8892
Peripheral vascular disease	1364 (3.5%)	1334 (3.5%)	1281 (3.7%)	1313 (3.4%)	5292 (3.5%)	0.2367
Psychoses	196 (0.5%)	203 (0.5%)	200 (0.6%)	177 (0.5%)	776 (0.5%)	0.2075
Pulmonary circulation disorders	524 (1.3%)	453 (1.2%)	470 (1.3%)	459 (1.2%)	1906 (1.3%)	0.0622
Renal disease	1484 (3.8%)	1478 (3.8%)	1412 (4.0%)	1572 (4.1%)	5946 (3.9%)	0.1448
Rheumatic disease	381 (1.0%)	398 (1.0%)	382 (1.1%)	365 (0.9%)	1526 (1.0%)	0.1617
Solid tumor without metastasis	3222 (8.2%)	3265 (8.4%)	3265 (9.3%)	3185 (8.2%)	12937 (8.5%)	<0.001
Valvular disease	829 (2.1%)	824 (2.1%)	788 (2.2%)	860 (2.2%)	3301 (2.2%)	0.5703
Weight loss	6027 (15.4%)	5977 (15.5%)	5900 (16.8%)	6109 (15.8%)	24013 (15.8%)	<0.001
Mortality, N (%)	1519 (3.9%)	1422 (3.7%)	1457 (4.2%)	1519 (3.9%)	5917 (3.9%)	0.0176
Intensive care stays, N (%)	3406 (8.7%)	3599 (9.3%)	3478 (9.9%)	3520 (9.1%)	14 003 (9.2%)	<0.001
Reoperation, N (%)	3424 (8.8%)	3391 (8.8%)	3308 (9.4%)	3301 (8.5%)	13 424 (8.9%)	<0.001

In order to account for patient clustering within hospitals, inpatient stays characteristics were compared between all 4 quarters using Rao-Scott chi-square tests for categorical variables and linear GEE regression models for continuous variables.

* Other diagnoses included all diagnoses present in colorectal surgery that did not fall into the other presented categories. The 10 more frequent other diagnoses included complete uterovaginal prolapse, rectal prolapse, acute peritonitis, acute vascular disorders of intestine, cystocele, endometriosis of intestine, rectocele, fistula of intestine, perforation of intestine (nontraumatic) and uterovaginal prolapse, unspecified.

**Fig 2:**
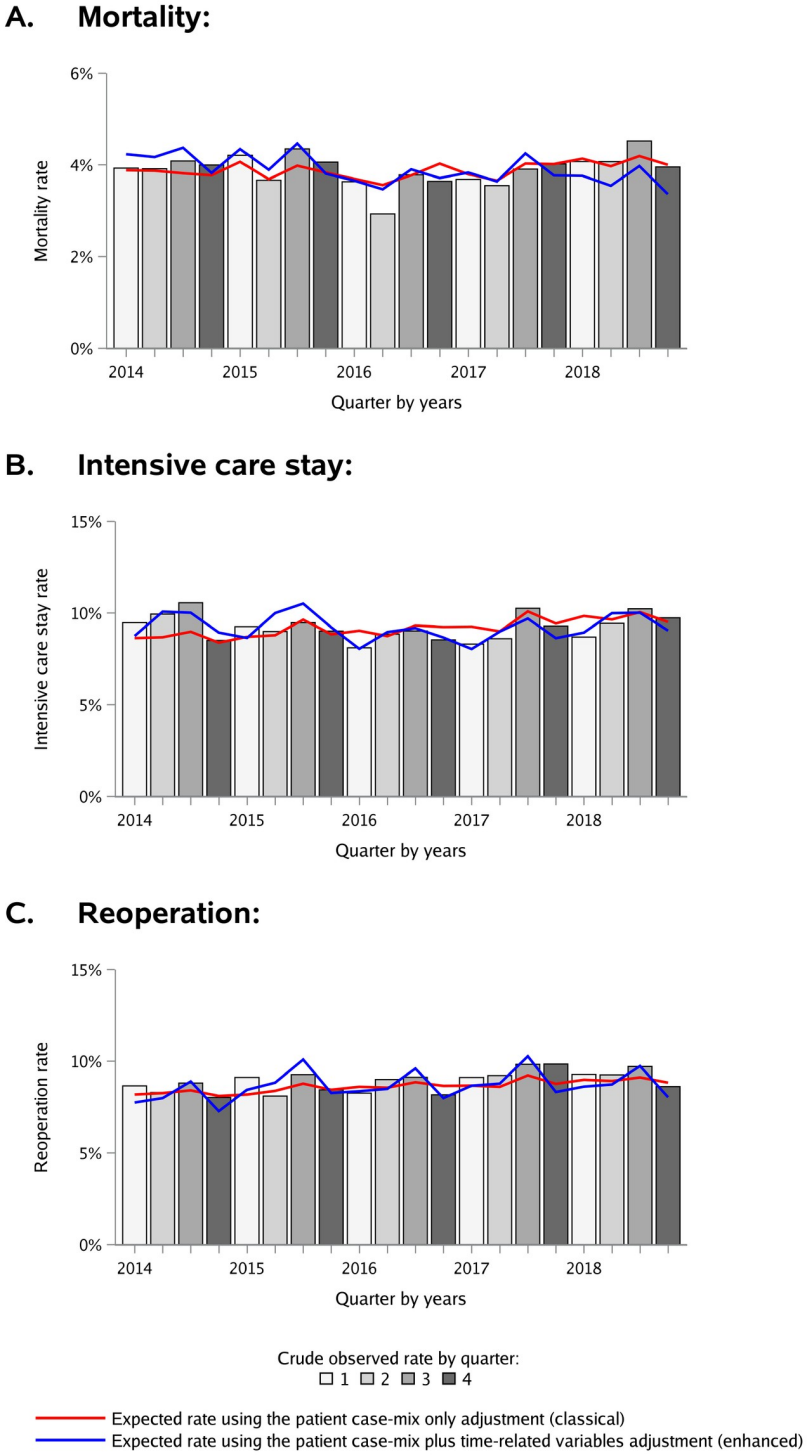
Histograms of observed outcomes and the related curves of expected trends with classical or enhanced adjustments. Expected rates of complications per quarter were calculated from the GEEs models initially fitted on the training dataset and then applied to the testing dataset. Models used for the construction of classical control charts were adjusted for case-mix variables only (age, gender, socioeconomic status, medical accessibility, emergency admission, hospital status, surgical procedure complexity, primary diagnosis, and comorbidities in dummies from the Elixhauser score). Models used for the construction of enhanced case-mix and time adjusted control charts were adjusted with the same set of variables, in addition to the year as a proxy for secular trends and the quarter as a proxy of seasonal variations.

**Fig 3:**
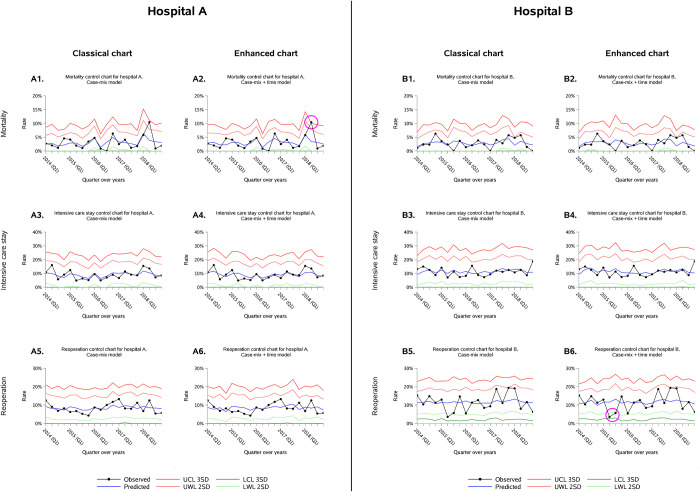
Example of classical and enhanced control charts for two hospitals. Crude observed rates (dotted black line) were monitored over 20 quarters for all three surgical outcomes. 2-SD warning limits (light green/red lines) and 3-SD control limits (bold green/red lines) were based on the central line (blue line) computed through the GEE models. A special cause variation related to a deterioration of surgical outcomes was detected in case of one single point beyond the 3-SD upper control limit (3-SD UCL), or 2 out of 3 consecutive points beyond the 2-SD upper warning limit (2-SD UWL). Conversely, a special cause variation related to an improvement of surgical outcomes was detected in case of one single point below the 3-SD lower control limit (3-SD LCL), or 2 out of 3 consecutive points below the 2-SD lower warning limit (2-SD LWL). The signal detection was considered at the first point beyond the limit when using the 2 out of 3 consecutive points rule. The two selected hospitals demonstrated discordances (encircled in pink) in interpretation of surgical outcome variations between classical and enhanced charts. Hospital A detected a special cause variation of increased mortality during the second quarter of 2018 using the time-adjusted chart (1 point above the 3-SD upper control limit, chart A2) but not the case-mix only adjusted chart (chart A1). Similarly, hospital B detected a special cause variation of decreased reoperation rate during the second quarter of 2015 using the time-adjusted chart (2 points out of 3 below the 2-SD lower warning limit, chart B6) but not the classical one (only 1 point out of 3 below the 2-SD warning limit, chart B5).

Models used to build the classical and the enhanced charts had discrimination (using C-statistics) ranging from 0.904 (95% Confidence Interval [95% CI], 0.896 to 0.911) for mortality to 0.812 (95% CI, 0.804 to 0.820) for intensive care stay and 0.715 (95% CI, 0.705 to 0.725) for reoperation. Model calibration was excellent, with a calibration line close to the diagonal for all models (see S3 Supporting information in S1 File). Fig 2 presents nationwide variations of surgical outcomes with iterative peak of expected deaths, intensive care stays and reoperations over the third quarter every year. The enhanced control charts considered those peaks in adjustment, providing a different interpretation of special cause variations compared to the classical control charts adjusted for patient case-mix only. Examples of classical and enhanced control charts discrepancies are displayed for two hospitals in Fig 3. In both cases the classical control charts did not detect special cause variations related to deterioration or improvement in surgical outcomes compared to the enhanced control charts.

Overall, among 59 signals detection of special cause variations, 19 discordances (32.2%) were observed between classical and enhanced charts, including 4 discordances for mortality, 6 for intensive care stay and 9 for reoperation. These discordances were mostly observed in the second and third quarters (6 discordances each), followed by the fourth (4) and the first quarters (3). There were 2 discordances in 2014, 6 in 2015, 2 in 2016, 4 in 2017, and 5 in 2018.

Using the classical chart, 9.5% (14/148) of hospitals detected at least one special cause variation for mortality along the 5-years monitoring period, 4.7% (7/148) for intensive care stay and 7.4% (11/148) for reoperation. Corresponding numbers with the enhanced chart were 9.5% (14/148), 4.7% (7/148) and 8.1% (12/148), respectively. Observed kappa agreement between classical and enhanced charts for detecting special cause variation in Table 2 was 0.89 for mortality (95% CI, 0.78 to 1.00), 0.83 for intensive care stay (95% CI, 0.70 to 0.96), and 0.67 for reoperation (95% CI, 0.46 to 0.87). Considering the enhanced time-adjusted chart as the reference, sensitivity and positive predictive values of the classical chart to detect special causes variations were both 0.89 (95% CI, 0.65 to 0.99) for inpatient mortality and 0.83 (95% CI, 0.59 to 0.96) for intensive care stay, and respectively 0.75 (95% CI, 0.43 to 0.95) and 0.60 (95% CI, 0.32 to 0.84) for reoperations.

**Table 2:** Comparison of special cause variations detection between classical and enhanced control charts.

		Enhanced case-mix and time-adjusted control chart		Cohen’s Kappa	Classical control chart vs Enhanced control chart (reference)	
		Signal	No signal		Sensitivity Value	Positive Predictive Value
Classical case-mix adjusted only control chart	**30-days in patient mortality: 4 discordances**					
	Signal	16	2	0.89 (0.78–1.00)	0.89 (0.65–0.99)	0.89 (0.65–0.99)
	No signal	2	2,940			
	**Intensive care stays: 6 discordances**					
	Signal	15	3	0.83 (0.70–0.96)	0.83 (0.59–0.96)	0.83 (0.59–0.96)
	No signal	3	2,939			
	**30-days reoperations: 9 discordances**					
	Signal	9	6	0.67 (0.46–0.87)	0.75 (0.43–0.95)	0.60 (0.32–0.84)
	No signal	3	2942			
	**Total: 19 discordances**					
	Signal	40	11	0.81 (0.72–0.89)	0.83 (0.70–0.93)	0.78 (0.65–0.89)
	No signal	8	8821			

## Discussion

### Main findings

Because variations in patient case-mix and data validity over time may bias interpretation of continuous surgical outcomes monitoring, we developed a control chart adjusted for secular and seasonal trends. Signal detection of special cause variations based on this enhanced chart was compared against a classical control chart that only considered patient case-mix. Although the number of special cause variations detecting better or worse surgical outcomes were similar for the classical and the enhanced control charts, a third of special cause variations were discordant. Indeed, a significant number of special cause variations were detected, whether rightly or wrongly, by one chart but not the other one. Under the assumption that the enhanced chart might have outperformed the classical chart since it considered more parameters, it would have detected not only more true positive signals but also less false positives. Omitting time-related proxies in adjustment schemes could lead to consider common cause as special cause variations, hence potentially undertaking inadequate interventions. Conversely, it could also lead to consider special cause as common cause variations, hence missing opportunities to improve care.

### Comparison with other studies

Statistical process control (SPC) tools such as control charts have already been applied to surgery and have proven to be useful in enhancing patient safety [2]. Because the mix of every patient and surgical procedure is different, adjusting SPC tools has been proposed to allow a more accurate interpretation of surgical outcome variations [26, 27]. Numerous studies have shown clear advantages in adjusting models comparatively to non-risk-adjusted tools [28] using validation methodologies similar to ours [25, 26]. A systematic review found that half of studies using control charts in surgery considered outcomes adjustment [6]. However, adjustment is complex and requires caution with the employed methodology and data [29–31]. Controversy exists over whether administrative data granularity and validity are sufficient to control potential confounders accurately [32]. Key variables might be unavailable, missing or inaccurate to measure case-mix variations directly.

Using proxies built from other available variables represents a solution to better capture case-mix indirectly, however. Seasonality and secular trends are frequently considered in the study of interrupted time series, but have not yet been used to build control charts [33]. Seasonality based on quarter as time period measurement can be considered as a useful proxy reflecting unknown case-mix parameters. Past studies have highlighted a greater concentration of severe cases during the summer [34], and deterioration in surgical outcomes due to the postponement of elective care and differences in staffing composition [8, 9, 35]. Secular trends in surgical outcomes over years have also been frequently described [15, 36]. Their adjustment can significantly change results interpretation and revealed crucial to avoid erroneous signal detection not associated with special cause variation within a particular hospital but rather with long-term improvement or deterioration in care delivery nationwide [37, 38].

### Strengths and limitations

In the present study, we designed an enhanced control chart accounting for both secular trends and seasonal variations through the integration of the year and quarter of performed surgical procedure in the adjustment scheme. We extracted data from a nationwide inpatient sample, an approach that can be easily reproduced with similar diagnosis related-like data sources available in other countries [39]. By focusing on colorectal surgery, we selected a high-volume surgery with frequent adverse events identifiable from medico-administrative data. We also intended to reduce heterogeneity in patient case-mix and to characterize accurately patient severity and procedure complexity by using specific algorithms. Reproducing our methodology would be relevant for assessing time-adjusted control charts in other healthcare contexts and countries experiencing various patterns of seasonal variations and secular trends. We also ensured that changes measured in care safety reflected only intra-hospital variations and not inter-hospital variations by modelling outcomes using multi-level GEE logistic regression models to capture risks specific to each hospital, considering the hospital status, and recalibrating our control charts.

Several limitations should also be acknowledged. First, we compared signal detection between the two control charts, assuming the time-adjusted chart as the most valid one. However, the true gold standard would have been to investigate systematically every signal detected from both charts within each hospital. The retrospective and nationwide nature of the pursued work did not allow us to pursue this approach. Second, adjusting for seasonal variations categorizes all resulting fluctuations as common cause variations, potentially masking recurrent quality deteriorations during suboptimal conditions such as in the summer. Third, even if the hospitals and patients sample size was reasonably large, signals occurrence was scarce and the related number small to compute statistics. Proposed metrics such as Cohen’s Kappa coefficients, sensitivity or positive predictive values might have been influenced by such small numbers resulting in a limited accuracy. Cohen’s Kappa might also be too optimistic due to the very high numbers in non-detections. We were also exposed to traditional limitations inherent to the use of a hospital administrative database. This may have resulted in case-mix heterogeneity issues due to differences in coding practices between hospitals [40, 41]. We attempted to bypass such potential pitfalls by monitoring surgical outcomes within each hospital instead of benchmarking across hospitals, and by recalibrating our charts based on each hospital’s average performance over the whole study period. In addition, changes in national data coding rules or coding variations that may exist within individual hospitals over time was considered by integrating the year and quarter in adjustment scheme. Finally, the broad diversity in diagnoses present in the study led us to adopt a classification that may have lacked finesse, especially in regards to procedures that involved other surgical specialties.

### Policy implications

We built a novel system to monitor surgical outcomes nationwide within every French hospital over time using time-adjusted control charts. This approach goes beyond traditional approaches such as benchmarking, avoiding potentially misleading conclusions [4] and enabling a dynamic follow-up over time. The use of hospital administrative databases that were not initially intended for healthcare quality and safety assessment is controversial. Using a clinical registry such as the ACS-NSQIP would have provided more reliable information on patient comorbidities and outcomes [42, 43]. However, administrative claims data provides nationwide exhaustibility and are easily replicable worldwide at low cost, as clinical registries are more expensive to maintain and only provide a sample of the whole population. Registries also have their limitations since there will always exist unmeasured patient-related variables to influence outcomes independently of the quality of care.

Our study underlined significant discordances in signal detection between the classical case-mix adjusted control chart and the enhanced time-adjusted chart. Not considering seasonal variations and secular trends in outcome adjustment is a concern to allow a fair interpretation of hospital performance. Indeed, biased interpretation of control chart signals can lead to inadequate investigation of special cause variations. Wrongly ignoring a signal (i.e. a false negative) deprives surgical teams of resolving significant safety issue and represents a missing opportunity to improve healthcare delivery. Conversely, wrongly detecting a signal that only reflects random variation (i.e. false positive) will waste the time and energy of teams investigating an event that never happened, and potentially introduce irrelevant changes with undesirable variations in surgical practices. Seasonal variations and secular trends are easily available in medico-administrative data and should be systematically considered in risk adjustment when monitoring surgical outcomes over time to enhance control charts interpretation.

## Supporting information

S1 File (DOCX)
