# Supplementary material for: A time-adjusted control chart for monitoring surgical outcome variations
Source: PLoS One. 2024 May 15;19(5):e0303543. doi: 10.1371/journal.pone.0303543 (PMC11095702; doi:10.1371/journal.pone.0303543)
Supplement: S1 File — (DOCX) [file pone.0303543.s001.docx]

**S1. Generalized Estimating Equations models**

Population average models estimated by generalized estimating equations (GEE) are a frequent approach to estimate regression for repeated measures data. We used logistic regression models using GEEs for parameters with an exchangeable working correlation structure to accound for clustering of patients within each hospital. Generalized estimating equations are well described in the 2009 paper of Hubbard and van der Laan (*Non parametric population average models: deriving the form of approximative population average models using generalized estimating equations, U.C. Berkeley working paper series, 2009*):

Considering Y_ij_ the outcome and X_ij_ the associated covariates, the population average model can be modeled as:

$$\begin{aligned} E\left( Y_{ij}|X_{ij} \right)=m\left( X_{ij}|\beta\right)=g\left[ u\left( X_{ij} | \beta\right) \right]\#(1) \end{aligned}$$

Here, $g$ denotes the links function, $\beta$ the regression coefficients and $u$ a linear function constructed from the vector of covariates $X_{ij}$. In the GEE approach, we aim to propose the true parameters that ensures than the mean of the estimating function is 0.

The general form of this estimating function for regression parameters is:

$$\begin{aligned} D_{V}\left( X_{.j},Y_{.j} | \beta\right)=\frac{\partial m\left( X_{.j} | \beta\right)}{\partial\beta}V^{-1}\left\{ \varepsilon_{.j}\left( \beta\right) \right\}\varepsilon_{.j}\left( \beta\right)^{T} \#(2) \end{aligned}$$

$X_{.j}$ represents the design matrix for all observations around j, $Y_{.j}$ the vector of outcomes, $m\left( X_{.j} | \beta\right)$ a vector of regression functions, $\beta$ a vector of regression coefficients and $V$ the working correlation structure.

Since this is a consistent estimating function (i.e. the mean is zero with the right parameters$\beta$), Hubbard and van der Laan stated that:

$$\begin{aligned} \sum_{i=1}^{m} D_{\hat{V}}\left( X_{.j}Y_{.j} | \beta\right)=0\#(3) \end{aligned}$$

Since these estimators are asymptomatically linear, authors deduced that:

$$\begin{aligned} \beta_{m}-\beta=\frac{1}{m}\sum_{j=1}^{m} IC\left( X_{.j},Y_{.j} | \beta\right)+O_{p}\left( \frac{1}{\sqrt{m}} \right)\#(4) \end{aligned}$$

Where $\beta_{m}$ stands for the estimates on m observations, $IC$ for the influence curve (i.e. a standardized version of the estimating function) and $O_{p}\left( \frac{1}{\sqrt{m}} \right)$ corresponds to a term that nullifies as m increases.

Finally, authors deduce $IC$:

$$\begin{aligned} IC\left( X_{.j},Y_{.j} | \beta\right)=E\left\{ h\left( X_{.j} \right)\frac{\partial m\left( X_{.j} | \beta\right)}{\partial\beta^{T}} \right\}h\left( X_{.j} \right)\varepsilon_{.j}\left( \beta\right)^{T}\#(4) \end{aligned}$$

With $h\left( X_{.j} \right)=\frac{\partial m}{\partial\beta}\left( X_{.j} | \beta\right)_{{pxn}_{j}}^{T}V^{-1}\left\{ \varepsilon_{.j}(\beta) \right\}_{n_{j}xn_{j}}$.

**S2. Guidelines for the construction of the central line and the exact control limits**

Using the regression estimates of the classical control chart (adjustment for case-mix only) and the enhanced control chart (adjustment for trends in addition to case-mix), we were able to to compute for each operated patient of the testing dataset i the expected probability of each outcome: $x_{M_{1},i}$ (classical model) and $x_{M_{2},i}$ (enhanced model).

1. Central line

The central line value of the charts $\bar{p_{M,j}}$ was not constant and represented the expected proportion of complications per quarter j, with a recalibration to reflect the overall observed rate in the hospital:

Recalibration term

(= overall observed rate – overall expected rate)

$\begin{aligned} \bar{p_{M,j}}= \frac{\sum_{i=1}^{k_{j}} x_{M,i}}{k_{j}}+\left( \frac{\sum_{i=1}^{n} y_{i}}{n}-\frac{\sum_{i=1}^{n} x_{M,i}}{n} \right)\#(1) \end{aligned}$

*Here, xi and yi respectively denote the probability of adverse events and the corresponding observed value for observation i, j the considered quarter, k the number of observations at quarter j, n the overall number of observations in the hospital and M the considered model (classical or enhanced).*

1. Control and warning limits

Exact control and warning limits were set at 3 SD (99.73%) and 2 SD (95.45%) from the central line respectively using the exact binomial distribution^[[1]](#footnote-1)^. The cumulative distribution function of the binomial distribution can be expressed as:

$$\begin{aligned} F\left( x_{i},p,n_{i} \right)=P\left( X_{i}\leq x_{i} \right)= \sum_{k=0}^{x} \binom{n_{i}}{k}p^{k}\left( 1-p \right)^{n_{i}-k}\#(2 \end{aligned})$$

The 3SD control limits, upper and lower, were calculated as the first values of $x_{i}$ that satisfy respectively: $P\left( X_{i}\leq x_{i} \right)=\frac{1+0.9973}{2}$ (UCL) and $\left( X_{i}\leq x_{i} \right)=\frac{1-0.9973}{2}$ (LCL).

By extension, the upper and lower 2SD warning limits were calculated similarly, as the first $x_{i}$ values satisfying $P\left( X_{i}\leq x_{i} \right)=\frac{1+0.9945}{2}$ and $P\left( X_{i}\leq x_{i} \right)=\frac{1-0.9545}{2}$, respectively.

In practice, exact control limits were calculated as follow:

$$\begin{aligned} {UCL}_{M,3SD}=\bar{p_{M,j}}+\left[ \frac{quantile\left( binom,\frac{1+0.9973}{2},\bar{p_{M,j}},k_{j} \right)}{k_{j}}-\bar{p_{M,j}} \right]\#(3 \end{aligned})$$

$$\begin{aligned} {LCL}_{M,3SD}=\bar{p_{M,j}}-\left[ \bar{p_{M,j}}-\frac{quantile\left( binom,\frac{1-0.9973}{2},\bar{p_{M,j}},k_{j} \right)}{k_{j}} \right]\#(4 \end{aligned})$$

Control limits were additionally corrected through an inflation factor derived from the models intra-class correlation coefficients to account for the inflation of variance due to the pooling of patients within hospitals:

$$\begin{aligned} \bar{{UCL}_{M,3SD}}=\bar{p_{M,j}}+\left[ \frac{quantile\left( binom,\frac{1+0.9973}{2},\bar{p_{M,j}},k_{j} \right)}{k_{j}}-\bar{p_{M,j}} \right]\times\sqrt{1+(\frac{k_{j}-1}{m})\times{ICC}_{M}}\#(5) \end{aligned}$$

$$\begin{aligned} \bar{{LCL}_{M,3SD}}=\bar{p_{M,j}}-\left[ \bar{p_{M,j}}-\frac{quantile\left( binom,\frac{1-0.9973}{2},\bar{p_{M,j}},k_{j} \right)}{k_{j}} \right]\times\sqrt{1+\left( \frac{k_{j}-1}{m} \right)\times{ICC}_{M}}\#\left( 6 \right) \end{aligned}$$

Where m stands for the number of clusters (i.e. number of hospitals). The same rules applied for the warning limits, the only difference being the use of 2SD values instead of 3SD.

Finally, in cases where calculated values were beyond 1 or below 0 (values that are conceptually impossible), such limits were reset to 1 and 0, respectively.

**S3. Calibration assessment for GEE logistic regression models**


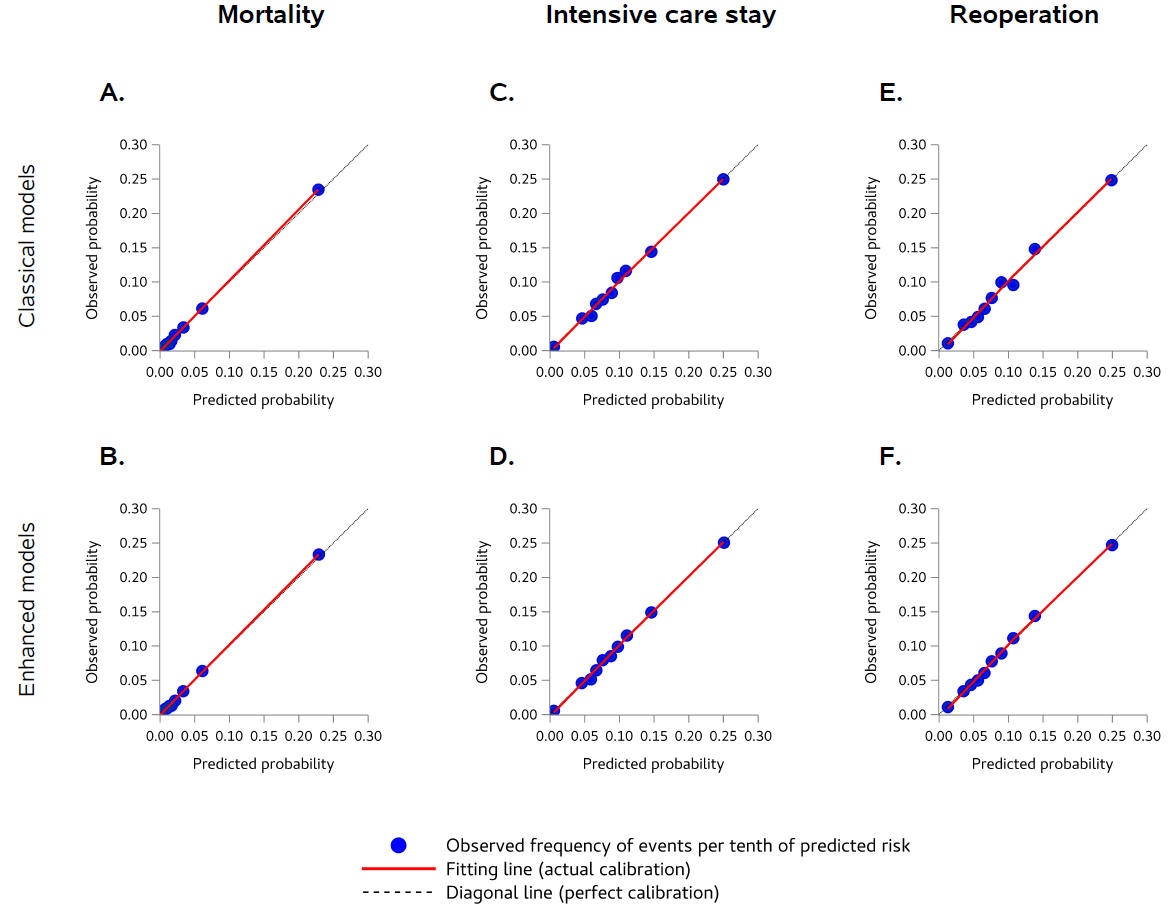


Model calibration was assessed by plotting the observed frequency of events per tenths of predicted risk.

**S4. The RECORD statement – checklist of items, extended from the STROBE statement, that should be reported in observational studies using routinely collected health data**

|  | **Item No.** | **STROBE items** | **Location in manuscript where items are reported** | **RECORD items** | **Location in manuscript where items are reported** |
| --- | --- | --- | --- | --- | --- |
| **Title and abstract** | | | | | |
|  | 1 | (a) Indicate the study’s design with a commonly used term in the title or the abstract (b) Provide in the abstract an informative and balanced summary of what was done and what was found | (a) Abstract (§Design)  (b) Abstract (§Methods, §Results and §Conclusion) | RECORD 1.1: The type of data used should be specified in the title or abstract. When possible, the name of the databases used should be included.  RECORD 1.2: If applicable, the geographic region and timeframe within which the study took place should be reported in the title or abstract.  RECORD 1.3: If linkage between databases was conducted for the study, this should be clearly stated in the title or abstract. | (1.1) Abstract (§Design)  (1.2) Region : abstract (§Setting)  Timeframe : abstract (§Participants)  (1.3) No linkage |
| **Introduction** | | | | | |
| Background rationale | 2 | Explain the scientific background and rationale for the investigation being reported | Introduction (§1-2-3 p4-5) |  |  |
| Objectives | 3 | State specific objectives, including any prespecified hypotheses | Introduction (§3 p5-6) |  |  |
| **Methods** | | | | | |
| Study Design | 4 | Present key elements of study design early in the paper | Methods (2.1 Study population, outcomes and design §3 p5-6) |  |  |
| Setting | 5 | Describe the setting, locations, and relevant dates, including periods of recruitment, exposure, follow-up, and data collection | Methods (2.1 Study population, outcomes and design §1 p5) |  |  |
| Participants | 6 | *(a) Cohort study* - Give the eligibility criteria, and the sources and methods of selection of participants. Describe methods of follow-up  *Case-control study* - Give the eligibility criteria, and the sources and methods of case ascertainment and control selection. Give the rationale for the choice of cases and controls  *Cross-sectional study* - Give the eligibility criteria, and the sources and methods of selection of participants  *(b) Cohort study* - For matched studies, give matching criteria and number of exposed and unexposed  *Case-control study* - For matched studies, give matching criteria and the number of controls per case | (a) Methods (2.1 Study population, outcomes and design §1 p5)  (b) Not applicable | RECORD 6.1: The methods of study population selection (such as codes or algorithms used to identify subjects) should be listed in detail. If this is not possible, an explanation should be provided.  RECORD 6.2: Any validation studies of the codes or algorithms used to select the population should be referenced. If validation was conducted for this study and not published elsewhere, detailed methods and results should be provided.  RECORD 6.3: If the study involved linkage of databases, consider use of a flow diagram or other graphical display to demonstrate the data linkage process, including the number of individuals with linked data at each stage. | (6.1) Methods (2.1 Study population, outcomes and design §1 p5)  (6.2) Not applicable  (6.3) Not applicable |
| Variables | 7 | Clearly define all outcomes, exposures, predictors, potential confounders, and effect modifiers. Give diagnostic criteria, if applicable. | Methods (2.1 Study population, outcomes and design §2-3 p5-6) | RECORD 7.1: A complete list of codes and algorithms used to classify exposures, outcomes, confounders, and effect modifiers should be provided. If these cannot be reported, an explanation should be provided. | (7.1) Not applicable |
| Data sources/ measurement | 8 | For each variable of interest, give sources of data and details of methods of assessment (measurement).  Describe comparability of assessment methods if there is more than one group | Methods (2.2 §Data sources p6-7) |  |  |
| Bias | 9 | Describe any efforts to address potential sources of bias | Methods (2.3 Statistics and charts): use of a multilevel model to account for patients clustering within hospitals §1 p8, use of the ICC to reflect inflation in variance due to the clustering of patients with hospitals §1 p7-8, central line recalibration §2 p9 |  |  |
| Study size | 10 | Explain how the study size was arrived at | Methods (2.1 Study population, outcomes and design §1 p5), Figure1: flowchart |  |  |
| Quantitative variables | 11 | Explain how quantitative variables were handled in the analyses. If applicable, describe which groupings were chosen, and why | Not applicable |  |  |
| Statistical methods | 12 | (a) Describe all statistical methods, including those used to control for confounding  (b) Describe any methods used to examine subgroups and interactions  (c) Explain how missing data were addressed  (d) *Cohort study* - If applicable, explain how loss to follow-up was addressed  *Case-control study* - If applicable, explain how matching of cases and controls was addressed  *Cross-sectional study* - If applicable, describe analytical methods taking account of sampling strategy  (e) Describe any sensitivity analyses | (a) Methods (2.3 Statistics and charts): §1-2-3 p7-9  (b) Methods (2.3 Statistics and charts): §1-2-3 p7-9 (c) Methods (2.1 Study population, outcomes and design §1 p5), Figure1 - flowchart : exclusion of 1,038 stays with missing socioeconomic data  (d) Methods (2.2 Data sources p6-7): no loss to follow-up  (e) None |  |  |
| Data access and cleaning methods |  | .. |  | RECORD 12.1: Authors should describe the extent to which the investigators had access to the database population used to create the study population.  RECORD 12.2: Authors should provide information on the data cleaning methods used in the study. | (12.1) Methods (2.2 Data sources p6-7)  (12.2) Methods (2.2 Data sources p6-7), Figure1 (flowchart) |
| Linkage |  | .. |  | RECORD 12.3: State whether the study included person-level, institutional-level, or other data linkage across two or more databases. The methods of linkage and methods of linkage quality evaluation should be provided. | (12.3) Not applicable |
| **Results** | | | | | |
| Participants | 13 | (a) Report the numbers of individuals at each stage of the study (*e.g.*, numbers potentially eligible, examined for eligibility, confirmed eligible, included in the study, completing follow-up, and analysed)  (b) Give reasons for non-participation at each stage.  (c) Consider use of a flow diagram | (a, b, c) Results (§1 p9-10), Figure1 (flowchart) | RECORD 13.1: Describe in detail the selection of the persons included in the study (*i.e.,* study population selection) including filtering based on data quality, data availability and linkage. The selection of included persons can be described in the text and/or by means of the study flow diagram. | (13.1) Methods (2.1 Study population, outcomes and design §1 p5-6), Figure1 (flowchart) |
| Descriptive data | 14 | (a) Give characteristics of study participants (*e.g.*, demographic, clinical, social) and information on exposures and potential confounders  (b) Indicate the number of participants with missing data for each variable of interest  (c) *Cohort study* - summarise follow-up time (*e.g.*, average and total amount) | (a) Results (§1 p9-10), Table1  (b) Methods (2.1 Study population, outcomes and design) p5-6 Figure1 (flowchart): 1,038 stays with missing socio-economic data retrieved from the analysis  (c) Not relevant |  |  |
| Outcome data | 15 | *Cohort study* - Report numbers of outcome events or summary measures over time  *Case-control study* - Report numbers in each exposure category, or summary measures of exposure  *Cross-sectional study* - Report numbers of outcome events or summary measures | Results (§1 p9-10),  Table 1 |  |  |
| Main results | 16 | (a) Give unadjusted estimates and, if applicable, confounder-adjusted estimates and their precision (e.g., 95% confidence interval). Make clear which confounders were adjusted for and why they were included  (b) Report category boundaries when continuous variables were categorized  (c) If relevant, consider translating estimates of relative risk into absolute risk for a meaningful time period | (a) Done  (b) Done  (c) Not relevant |  |  |
| Other analyses | 17 | Report other analyses done—e.g., analyses of subgroups and interactions, and sensitivity analyses | None |  |  |
| **Discussion** | | | | | |
| Key results | 18 | Summarise key results with reference to study objectives | Discussion (§1 p16) |  |  |
| Limitations | 19 | Discuss limitations of the study, taking into account sources of potential bias or imprecision. Discuss both direction and magnitude of any potential bias | Discussion (§1 p16) | RECORD 19.1: Discuss the implications of using data that were not created or collected to answer the specific research question(s). Include discussion of misclassification bias, unmeasured confounding, missing data, and changing eligibility over time, as they pertain to the study being reported. | Discussion (§4 p17-18) |
| Interpretation | 20 | Give a cautious overall interpretation of results considering objectives, limitations, multiplicity of analyses, results from similar studies, and other relevant evidence | Discussion (§2 p16-17) |  |  |
| Generalisability | 21 | Discuss the generalisability (external validity) of the study results | Discussion (§3 p19-20) |  |  |
| **Other Information** | | | | | |
| Funding | 22 | Give the source of funding and the role of the funders for the present study and, if applicable, for the original study on which the present article is based | Funding section  (p24) |  |  |
| Accessibility of protocol, raw data, and programming code |  | .. |  | RECORD 22.1: Authors should provide information on how to access any supplemental information such as the study protocol, raw data, or programming code. | Data sharing statement  (p6-7) |

*Reference: Benchimol EI, Smeeth L, Guttmann A, Harron K, Moher D, Petersen I, Sørensen HT, von Elm E, Langan SM, the RECORD Working Committee. The REporting of studies Conducted using Observational Routinely-collected health Data (RECORD) Statement. *PLoS Medicine* 2015; in press.

*Checklist is protected under Creative Commons Attribution ([CC BY](http://creativecommons.org/licenses/by/4.0/)) license.

1. *Duclos A, Voirin N. The p-control chart: a tool for care improvement. Int J Qual Health Care J Int Soc Qual Health Care. 2010 Oct;22(5):402–7.* [↑](#footnote-ref-1)
